# The transcribed ultraconserved regions: emerging players in colorectal cancer biology and therapy

**DOI:** 10.3389/fcell.2025.1599891

**Published:** 2026-01-02

**Authors:** Yi Zhang, Zichen Wei, Xin Wang, Pan Wang, Lei Pang, Hongliang Dong, Han Xu

**Affiliations:** 1 Department of Clinical Pharmacy, The People’s Hospital of Jiaozuo, Jiaozuo, Henan, China; 2 Yangzhou University Medical College, Yangzhou, Jiangsu, China; 3 Department of Pathology, Dongtai Hospital of Traditional Chinese Medicine, Dongtai, Jiangsu, China; 4 Department of Pathology, The First People’s Hospital of Linping District, Hangzhou, Zhejiang, China; 5 Department of Pediatrics Second Ward, The People’s Hospital of Jiaozuo, Jiaozuo, Henan, China; 6 Department of Gastroenterology, The Affiliated Hospital of Yangzhou University, Yangzhou University, Yangzhou, Jiangsu, China

**Keywords:** colorectal cancer, molecular mechanism, Transcribed ultraconserved regions, T-UCRs, tumor

## Abstract

Colorectal cancer (CRC) is one of the most prevalent malignant neoplasms worldwide, characterized by a high incidence of recurrence and metastasis, which substantially diminishes patient survival rates. This underscores the urgent need to identify novel biomarkers and therapeutic targets. Transcribed ultraconserved regions (T-UCRs), a category of non-coding RNAs with significant evolutionary conservation, are crucial to various biological processes. Recent studies have shown that T-UCRs play a pivotal role in tumorigenesis and tumor progression. A growing body of evidence indicates that T-UCRs significantly influence CRC development by modulating critical mechanisms, including cell proliferation, apoptosis, invasion, and metastasis. This review systematically explores the functions of T-UCRs in tumorigenesis, focusing on their regulatory roles, underlying molecular mechanisms, and potential clinical applications in CRC.

## 1 Introduction

The Global Burden of Disease study highlights that the incidence and mortality rates of CRC have doubled from 1990 to 2021, with projections indicating that CRC will continue to pose a substantial burden on global health systems by 2050 (Zhou et al., 2025; Matsuda et al., 2025). While the incidence of CRC in high-income countries have either stabilized or declined, largely due to enhanced screening practices, polypectomy in older populations, and changes in risk factors such as reduced smoking rates, early-onset CRC is rising among younger demographics (Keum and Giovannucci, 2019; Du et al., 2025; Li et al., 2023). Moreover, given that related symptoms frequently emerge at advanced stages, the identification and utilization of early diagnostic biomarkers are critically important.

**FIGURE 1:**
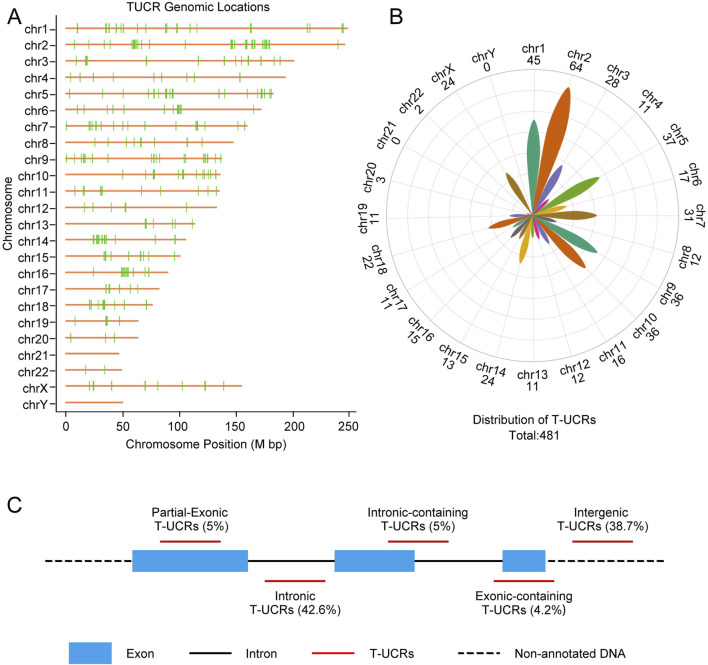
T-UCRs genomic locations and their classification. **(A,B)** Schematic representation of the genomic location and distribution of the 481T-UCRs. **(C)** Classification of the T-UCRs and its percentage. The image B was created using the ChiPlot tool (https://www.chiplot.online/).

Transcribed ultraconserved regions (T-UCRs), derived from ultraconserved regions (UCRs), exhibit high sequence conservation and tissue specificity, contributing significantly to cellular homeostasis and development. Previous study have identified 481 ultraconserved regions that are highly conserved across the genomes of various species and are distributed across most chromosomes (except chr21 and chrY), chr2 contains the highest number of UCRs (Bejerano et al., 2004) (Figures 1A,B). Another research subsequently refined this classification by examining the positional relationship of T-UCRs with genes, identifying five distinct types: exonic-containing (4.2%), intronic-containing (5%), partial-exonic (5%), intergenic (38.7%), and intronic (42.6%) (Mestdagh et al., 2010) (Figure 1C). Each T-UCR can produce transcripts from its sense and/or antisense strands, culminating in a total of 962 potential transcripts (Hudson et al., 2013).

T-UCRs play a significant role in various biological processes, including gene regulation, chromatin remodeling, and genomic stability maintenance. Aberrant expression of T-UCRs is strongly linked to a range of diseases, particularly cancer. Evidence suggests that T-UCRs serve as crucial regulatory elements in tumors like CRC, affecting cell proliferation, apoptosis, invasion, and metastasis. They also interact with established oncogenic and tumor-suppressive networks, contributing to a complex regulatory landscape (Kottorou et al., 2018; Sana et al., 2012; Zhang et al., 2018; Chen et al., 2024; Kuwano et al., 2021; Kottorou et al., 2020). Consequently, T-UCRs present substantial potential as diagnostic and prognostic biomarkers, as well as therapeutic targets for CRC.

Building upon the established link between T-UCR dysregulation and cancer, this review aims to systematically elucidate the multifaceted roles of T-UCRs in CRC pathogenesis. We first consolidate evidence detailing how specific T-UCRs govern core tumor cell-intrinsic phenotypes, including proliferation, apoptosis, and metastasis. Subsequently, we dissect the underlying molecular mechanisms, such as CpG island methylation and miRNA interactions, through which T-UCRs exert these functions. Furthermore, we expand the discussion beyond the cancer cell by exploring the emerging concept of T-UCRs as modulators of the tumor microenvironment. Finally, by integrating these layers of evidence, we critically evaluate the translational potential of T-UCRs as novel biomarkers and therapeutic targets, aiming to identify precise knowledge gaps that can guide future research in CRC biology and therapy.

## 2 T-UCRs in cancer research

**FIGURE 2:**
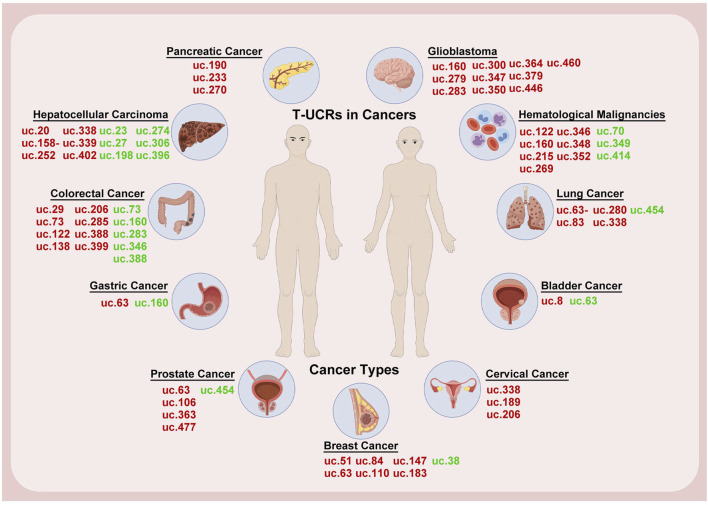
T-UCRs in cancer research. Changes in the expression of T-UCRs are illustrated in various human cancer types. Sex-specific cancers are shown below the male and female icons. Overexpression is shown in red and downregulation is shown in green. The image was created using the Figdraw 2.0 (https://www.figdraw.com/).

T-UCRs were discovered in 2004 and found in 2007 to be mainly located at genomic fragile sites linked to cancer, showing a strong association with human chronic lymphocytic leukemia (CLL) and colorectal cancer (CRC) (Calin et al., 2007). Further research indicates that T-UCRs are crucial in prostate cancer pathogenesis, affecting Gleason scores, extraprostatic extension, and treatment responses (Mestdagh et al., 2010; Hudson et al., 2013). The unique expression patterns of T-UCRs in different tumors offer promising biomarkers for early diagnosis and prognosis. We have compiled and highlighted T-UCRs that are closely associated with specific cancers, supported by differential expression data across various cancers (Figure 2; Table 1).

**TABLE 1:** T-UCRs in cancer research.

Cancers	T-UCRs	Role in disease	Expression	References
Hematological malignancies	uc.112	Promote	Upregulated	Calin et al. (2007), das Chagas et al. (2020), Ding et al. (2021)
	uc.160	Promote	Upregulated	
	uc.215	Promote	Upregulated	
	uc.269	Promote	Upregulated	
	uc.346	Promote	Upregulated	
	uc.348	Promote	Upregulated	
	uc.352	Promote	Upregulated	
	uc.70	Suppress	Downregulated	
	uc.349	Suppress	Downregulated	
	uc.414	Suppress	Downregulated	
Colorectal cancer	uc.29	Promote	Upregulated	Kottorou et al. (2018), Sana et al. (2012), Zhang et al. (2018), Chen et al. (2024), Kuwano et al. (2021), Kottorou et al. (2020), Calin et al. (2007)
	uc.122	Promote	Upregulated	
	uc.138	Promote	Upregulated	
	uc.206	Promote	Upregulated	
	uc.285	Promote	Upregulated	
	uc.399	Promote	Upregulated	
	uc.160	Suppress	Downregulated	
	uc.283	Suppress	Downregulated	
	uc.346	Suppress	Downregulated	
	uc.73	Promote/Suppress	Up/Downregulated	
	uc.388	Promote/Suppress	Up/Downregulated	
Gastric cancer	uc.63	Promote	Upregulated	Honma et al. (2017), Pang et al. (2018), Sakamoto et al. (2020)
	uc.160	Suppress	Downregulated	
Hepatocellular carcinoma	uc.20	Promote	Upregulated	Calin et al. (2007), Bo et al. (2016), Carotenuto et al. (2016), Luo et al. (2017), Kogure et al. (2013), Braconi et al. (2010)
	uc.158-	Promote	Upregulated	
	uc.252	Promote	Upregulated	
	uc.338	Promote	Upregulated	
	uc.339	Promote	Upregulated	
	uc.402	Promote	Upregulated	
	uc.23	Suppress	Downregulated	
	uc.27	Suppress	Downregulated	
	uc.198	Suppress	Downregulated	
	uc.274	Suppress	Downregulated	
	uc.306	Suppress	Downregulated	
	uc.396	Suppress	Downregulated	
Breast cancer	uc.51	Promote	Upregulated	Corrà et al. (2021), Pereira Zambalde et al. (2021), Marini et al. (2017), Shi et al. (2021), Zhang et al. (2017)
	uc.63	Promote	Upregulated	
	uc.84	Promote	Upregulated	
	uc.110	Promote	Upregulated	
	uc.147	Promote	Upregulated	
	uc.183	Promote	Upregulated	
	uc.38	Suppress	Downregulated	
Lung cancer	uc.63-	Promote	Upregulated	Gao et al. (2016), Vannini et al. (2022), Liu et al. (2020), Zhou et al. (2018)
	uc.83	Promote	Upregulated	
	uc.280	Promote	Upregulated	
	uc.338	Promote	Upregulated	
	uc.454	Suppress	Downregulated	
Prostate cancer	uc.63	Promote	Upregulated	Hudson et al. (2013), Sekino et al. (2017)
	uc.106	Promote	Upregulated	
	uc.363	Promote	Upregulated	
	uc.477	Promote	Upregulated	
	uc.454	Suppress	Downregulated	
Bladder cancer	.uc.8	Promote	Upregulated	Terreri et al. (2021), Olivieri et al. (2016)
	uc.63	Promote	Upregulated	
Cervical cancer	uc.338	Promote	Upregulated	Li et al. (2017), Wang et al. (2017), Li et al. (2016)
	uc.189	Promote	Upregulated	
	uc.206	Promote	Upregulated	
Glioblastoma	uc.160	Promote	Upregulated	Mestdagh et al. (2010), Watters et al. (2013), Soler et al. (2021), Galasso et al. (2014)
	uc.279	Promote	Upregulated	
	uc.283	Promote	Upregulated	
	uc.300	Promote	Upregulated	
	uc.347	Promote	Upregulated	
	uc.350	Promote	Upregulated	
	uc.364	Promote	Upregulated	
	uc.379	Promote	Upregulated	
	uc.446	Promote	Upregulated	
	uc.460	Promote	Upregulated	
Pancreatic cancer	uc.190	Promote	Upregulated	Jiang et al. (2016)
	uc.233	Promote	Upregulated	
	uc.270	Promote	Upregulated	

### 2.1 Hematological malignancies

Recent studies have shown increased interest in the role of T-UCRs in blood cancers. Research on pediatric bone marrow samples indicates that uc.112 expression is significantly higher in T-cell acute lymphoblastic leukemia (T-ALL) compared to B-cell acute lymphoblastic leukemia (B-ALL), with no notable differences in non-tumor T and B precursor cells. Moreover, uc.112 is upregulated in hyperploid B-ALL patients, underscoring its significance in pediatric ALL (das Chagas et al., 2020).

In CLL, nineteen T-UCRs, such as uc.349 and uc.352, serve as biomarkers to differentiate tumor from non-tumor samples and are located in the 13q21.33-q22.2 chromosomal region linked to familial CLL (Ding et al., 2021). Another study found significant downregulation of uc.70 and uc.414 in six primary CLL cases, confirmed in twelve more cases (Calin et al., 2007). These observations indicate that T-UCRs may play a substantial role in the pathogenesis of hematological malignancies, presenting potential novel targets for therapeutic intervention.

### 2.2 Colorectal cancer

Recent studies on T-UCRs in CRC reveal their crucial roles in cancer development and progression. Evidence indicates that T-UCRs are usually abnormally expressed in CRC tissues, with increased methylation levels, implying important regulatory functions. These methylation profiles may serve as potential biomarkers for non-invasive colorectal cancer screening (Kottorou et al., 2018).

Research has identified that uc.29A, uc.112, uc.206, and uc.399A show high expression in CRC, while uc.73 has inconsistent expression pattern (Calin et al., 2007). The uc.73A has been reported as one of the most significantly upregulated T-UCRs in CRC, yet it has also been noted that uc.73 is significantly downregulated, which may be attributed to the different mechanisms of action of the positive and negative strands (Sana et al., 2012; Calin et al., 2007). Another research found that uc.388 is downregulated in CRC tumors, with its reduced expression associated with distant metastasis (Sana et al., 2012). Typically, uc.338 is upregulated in CRC, and its expression is linked to tumor size, invasion depth, and lymph node metastasis (Zhang et al., 2018). Recent studies show that uc.285+ stabilizes CDC42 mRNA and protein, promoting the proliferation of CRC cells (Chen et al., 2024). Additionally, uc.138 is found to be upregulated in CRC cells, boosting cell proliferation and migration (Kuwano et al., 2021).

In chemotherapy-resistant HT-29 cells, uc.160, uc.283, and uc.346 were significantly downregulated, with uc.283 and uc.346 exhibiting similar patterns in cells resistant to oxaliplatin (Kottorou et al., 2020). These results highlight the importance of T-UCRs expression and methylation changes in CRC, suggesting new avenues for early diagnosis and treatment.

### 2.3 Gastric cancer

Research on gastric cancer has identified uc.160+ as significantly downregulated in gastric adenomas and gastric cancer tissues, linked to increased DNA methylation (Honma et al., 2017). This indicates a possible involvement of T-UCRs in gastric cancer via epigenetic regulation, based on current limited evidence. Our previous research aligns with these findings, revealing a significant downregulation of uc.160 in gastric cancer tissues. In gastric cancer cell lines, uc.160 enhances the tumor suppressor PTEN, thus inhibiting gastric cancer cell proliferation (Pang et al., 2018). Another study found that uc.63+ expression is significantly increased in gastric cancer tissues and is linked to advanced clinical features. Reducing uc.63+ expression hinders cancer cell growth and migration, whereas increasing it promotes these processes (Sakamoto et al., 2020).

### 2.4 Hepatocellular carcinoma

Hepatocellular carcinoma (HCC) is one of the most prevalent cancers globally. Emerging evidence indicates that T-UCRs are critically involved in the processes of malignant transformation, tumor proliferation, and metastasis associated with HCC. Specifically, uc.20, uc.252, and uc.402 are increased, while uc.23, uc.27, uc.198, uc.274, and uc.396 are decreased in HCC (Calin et al., 2007). Further investigations revealed that uc.338 expression was significantly increased in HCC cells, promoting cell proliferation by interacting with BMI1 (one component of multi comb group complex 1) to suppress p21 (Cell cycle dependent kinase inhibitor) expression, indicating a potential oncogenic role for uc.338 (Bo et al., 2016).

In addition, Uc.158- is upregulated in HCC due to Wnt/β-catenin pathway activation (Carotenuto et al., 2016). In HBV-related HCC, uc.306 expression is lower in tumors than in nearby healthy tissues, and this diminished expression was associated with poorer overall survival (Luo et al., 2017). Additionally, uc.339 was highly expressed in exosomes from HCC cells compared to expression in their donor cells, promoting tumor growth and adhesion through intercellular transfer (Kogure et al., 2013). In HepG2 cells, uc.338 levels were significantly higher than in non-cancerous liver cells. Blocking uc.338 increased G1/S transition genes like p16INK4a and decreased the activity of cyclin-dependent kinases CDK4 and CDK6 (Braconi et al., 2010). These insights reveal the biological functions of T-UCRs and identify novel targets, providing innovative concepts for future therapeutic interventions of HCC.

### 2.5 Breast cancer

In breast cancer (BC) research, uc.183, uc.110, and uc.84 inversely correlate with miR-221 and are implicated in the regulation of CDKN1B expression. Notably, altering these T-UCRs does not impact miR-221 levels after anti-cancer drug treatment, suggesting that specific T-UCRs may have substantial regulatory roles in breast cancer (Corrà et al., 2021). Furthermore, Uc.147 is significantly overexpressed in Luminal A and B breast cancer subtypes, with its upregulation in Luminal A linked to reduced survival (Pereira Zambalde et al., 2021). Similarly, uc.63 is overexpressed in Luminal A, associated with poor prognosis, and its inhibition induces apoptosis in breast cancer cells (Marini et al., 2017).

Additionally, uc.51, another significant T-UCR, exhibits increased expression in breast cancer, positively correlating with tumor size. The upregulation of uc.51 facilitates breast cancer cell proliferation, migration, and invasion via interaction with the non-POU domain-containing octamer-binding protein (NONO), which affects CREB phosphorylation (Shi et al., 2021). Conversely, uc.38 is downregulated in breast cancer, with lower levels associated with larger tumor size and more advanced cancer stages. Its upregulation may inhibit cell growth and induce apoptosis by downregulating PBX1, thus affecting the Bcl-2 family proteins (Bax and Bcl-2). Overexpression of PBX1 can increase the level of anti-apoptotic protein (Bcl-2) and reduce the level of pro-apoptotic protein (Bax) (Zhang et al., 2017). Collectively, Research on T-UCRs in breast cancer provides new insights into their roles in tumor biology.

### 2.6 Lung cancer

In lung cancer research, T-UCRs are biologically and clinically important. Specifically, uc.338 is highly expressed in lung cancer tissues, and its suppression reduces cancer cell growth, migration, and invasion. This also affects proteins related to the cell cycle and Epithelial-mesenchymal transition (EMT) (Gao et al., 2016). Additionally, a separate study found that uc.83- is highly upregulated in lung cancer, which markedly influences cell growth and proliferation. This oncogenic function is closely associated with the phosphorylation of AKT and ERK 1/2 (Vannini et al., 2022).

Research on Xuanwei lung cancer (LCXW) from Yunnan Province revealed that uc.63- and uc.280+ are also upregulated compared to paired adjacent noncancerous lung (NCL) tissues, correlating with tumor stage and prognosis (Liu et al., 2020). Besides, uc.454 is considered as a potential tumor suppressor due to its significantly reduced expression in NSCLC tissues and cell lines. This reduction could serve as an indicator of poor prognosis, given its role in negatively regulating the HSPA12B protein, affecting Bcl-2 family expression and inducing apoptosis (Zhou et al., 2018).

### 2.7 Prostate cancer

Research on T-UCRs in prostate cancer (PC) has revealed dysregulated expression of uc.106, uc.477, uc.363, and uc.454, with these expression patterns correlated with cancer progression, Gleason scores, and tumor extravasation (Hudson et al., 2013). Some T-UCRs are responsive to epigenetic drugs and androgens. In LnCap cells, uc.287 expression increases with androgen R1881, and uc.283+ rises with 5-Aza-2′-deoxycytidine and curcumin A treatment, suggesting their potential as targets in androgen receptor signaling or other epigenetic therapies (Hudson et al., 2013).

A separate study found that uc.63 expression is elevated in prostate cancer tissues, enhancing cell growth and migration. It influences MMP2 levels by regulating miR-130b, leading to increased resistance to docetaxel. Higher serum levels of uc.63 in patients treated with docetaxel were associated with drug resistance and survival rates (Sekino et al., 2017). Overall, T-UCRs are crucial in prostate cancer progression and resistance.

### 2.8 Bladder cancer

In bladder cancer (BlCa), uc.8 is significantly upregulated, correlating with tumor grade and stage. The translocation of uc.8+ from the nucleus to the cytoplasm facilitates its interaction with miR-596, suppressing YY1 and promotion of MMP9 expression, which increases cancer cell invasiveness (Terreri et al., 2021). Another study found that uc.8 shuttles from the nucleus to the BlCa cytoplasm and interacting with miR-596, resulting in increased expression of MMP-9 and increasing the invasive potential of BlCa cells (Olivieri et al., 2016). Additionally, uc.63 is overexpressed in bladder urothelial carcinoma tissues, increasing cisplatin resistance by affecting androgen receptor signaling (Sekino et al., 2019).

### 2.9 Other cancers

In cervical cancer (CC), the expression of uc.338 is significantly upregulated and is associated with lymph node metastasis. Moreover, uc.338 promotes cellular migration and invasion by targeting and downregulating the expression ofTIMP1 (Li et al., 2017). An analysis of 243 gynecological tumor samples found uc.189 was upregulated in 78.5% of cervical squamous cell carcinoma cases. Its overexpression was associated with poor prognosis and suggesting its potential as a prognostic biomarker for cervical and other gynecological tumors (Wang et al., 2017). Additionally, uc.206 is significantly upregulated in cervical cancer, indicating its potential role as an oncogene. The expression of uc.206 is inversely correlated with the pro-apoptotic gene TP53 by directly targeting its 3′UTR, thereby suppressing TP53 expression, promoting cancer cell proliferation, and inhibiting apoptosis (Li et al., 2016).

In glioblastoma, several T-UCRs like uc.347, uc.350, uc.279, uc.460, uc.379, uc.446, and uc.364, are significantly upregulated in MYCN-amplified tumors (Mestdagh et al., 2010). Treatment with all-trans retinoic acid (ATRA) can induce differentiation in neuroblastoma cells, reducing MYC transcription and notably impacting T-UCRs expression, especially uc.300 exhibiting the most pronounced decrease. This indicates a possible association between ATRA treatment and the downregulation of uc.300 (Watters et al., 2013). Moreover, higher methylation of the uc.160 CpG island is independently associated with improved survival outcomes in patients with low-grade gliomas. Mechanistically, uc.160 affects the downstream genes regulated by miR-376, including RYBP and FOXP2 (Soler et al., 2021). Additionally, uc.283 is significantly overexpressed in gliomas, although its molecular mechanisms remain to be fully elucidated (Galasso et al., 2014).

In the context of pancreatic cancer, uc.190, uc.233, and uc.270 are consistently upregulated. Inhibiting these T-UCRs reduces precursor PDAC cell proliferation, indicating their regulatory role in PDAC development (Jiang et al., 2016). Additionally, The transcription factor EGR1 appears to regulate T-UCRs expression, as its absence significantly reduces the level of these T-UCRs (Jiang et al., 2016).

## 3 The regulatory role of T-UCRs in CRC

**FIGURE 3:**
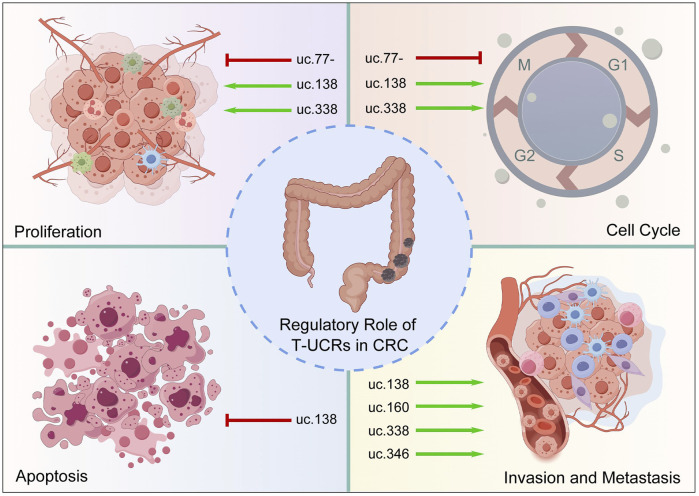
The regulatory role of T-UCRs in CRC. The functions of T-UCRs in CRC cellular proliferation, cell cycle, apoptosis, and their role in tumor invasion and metastasis. Green arrows indicate the promotion, and red arrows indicate the suppression. The image was created using the Figdraw 2.0 (https://www.figdraw.com/).

T-UCRs are integral to the regulation of CRC pathogenesis and progression. This section explores the diverse functions of T-UCRs in CRC, focusing on their influence on cellular proliferation, cell cycle, apoptosis, and their role in tumor invasion and metastasis (Figure 3).

### 3.1 T-UCRs in the proliferation and cell cycle of CRC cells

Cell proliferation must maintain a dynamic equilibrium to preserve tissue homeostasis and prevent malignant transformation. Aberrant cell proliferation is a fundamental characteristic of tumorigenesis, and the dysregulated expression of T-UCRs in CRC is recognized as a critical factor contributing to the disruption of this equilibrium (Kottorou et al., 2018; Zhang et al., 2018; Chen et al., 2024; Kuwano et al., 2021). Cell cycle regulation is a critical process underlying the proliferation of cancer cells. T-UCRs have the potential to modulate cell cycle progression in CRC cells through multiple pathways, influencing their proliferative capacity (Zhang et al., 2018; Kuwano et al., 2021; Zheng et al., 2021).

Specifically, research indicates that overexpression of uc.138 significantly enhances the proliferation of CRC cells, primarily by upregulating cell cycle-related genes such as CDK1, cyclin A, and cyclin B, while concurrently downregulating the cyclin-dependent kinase inhibitor p21 (CDKN1A), particularly during the G2 and S phases. This regulatory imbalance results in accelerated cell division and proliferation of cancer cells (Kuwano et al., 2021). Conversely, uc.77- exhibits an inhibitory effect on the proliferation of CRC cells. Its overexpression markedly suppresses both proliferation and growth capabilities of HCT116 and SW620 cell lines. *In vivo* studies reveal that uc.77- overexpression significantly reduces tumor growth in a nude mouse xenograft model, suggesting its antitumor effects through inhibition of cell proliferation. Meanwhile, uc.77- inhibits CRC cell proliferation by competitively binding to miR-4676-5p and negatively regulating CDK4, thereby indicating its suppressive role in cell cycle regulation (Zheng et al., 2021). Similarly, knockdown of uc.338 impedes the proliferation of LoVo and HCT116 cells by decreasing cell viability and DNA synthesis rates. Uc.338 facilitates the G1/S transition by downregulating p21 and upregulating cyclin D1, suggesting its oncogenic role in cell cycle regulation (Zhang et al., 2018).

In conclusion, T-UCRs play a critical role in regulating the proliferation of CRC cells through diverse mechanisms, which include not only the regulation of the cell cycle but also the modulation of key intracellular proteins, underscoring their significance in tumor proliferation.

### 3.2 T-UCRs in the apoptosis of CRC cells

Apoptosis is a crucial mechanism for maintaining cellular homeostasis, and cancer cells frequently acquire a proliferative advantage by inhibiting apoptotic pathways. T-UCRs play a significant role in the apoptosis of CRC cells, primarily through their regulation of apoptosis-related genes. Specifically, overexpression of uc.138 has been shown to downregulate genes associated with cell death, such as TP53, CASP2, CASP7, CASP9, RAS, BCL-XL, NFKB1 and so on, thereby reducing the sensitivity of cancer cells to chemotherapeutic agents such as 5-fluorouracil and doxorubicin. This modulation ultimately enhances the survival rate of cancer cells (Kuwano et al., 2021). This finding indicates that T-UCRs are integral not only to the proliferation of CRC cells but also to cancer progression by impeding apoptosis.

### 3.3 T-UCRs in the invasion and metastasis of CRC cells

Invasion and metastasis are key factors contributing to poor patient outcomes in CRC (Sun et al., 2021; Wang et al., 2018; Wang et al., 2023). Recent studies have demonstrated that T-UCRs significantly impact the progression of CRC by modulating cellular migration and invasion capabilities (Kottorou et al., 2018; Kuwano et al., 2021; Wang et al., 2016). Specifically, uc.138 has been shown to enhance the malignant phenotype of CRC by facilitating cell migration and promoting tumor growth, likely through the regulation of genes associated with cell motility and morphology (Kuwano et al., 2021). Besides, overexpression of uc.160 and uc.346 markedly increases the motility of CRC cells, further supporting their role in enhancing the invasive capabilities of these cells (Kottorou et al., 2018). Additionally, elevated expression of UC.338, identified as an oncogene, is significantly correlated with lymph node metastasis. Its downregulation has been shown to substantially inhibit the invasion and migration of cancer cells, underscoring its pivotal role in driving tumor metastasis (Wang et al., 2016).

### 3.4 T-UCRs in modulating the colorectal cancer tumor microenvironment

The pathogenesis and progression of colorectal cancer are orchestrated not only by malignant epithelial cells but also by the dynamic and interactive tumor microenvironment (TME). The CRC TME is a complex ecosystem comprising diverse immune cells, cancer-associated fibroblasts (CAFs), endothelial cells, signaling molecules, and a modified extracellular matrix (Teresa et al., 2025). This ecosystem plays a decisive role in tumor growth, immune evasion, metastasis, and therapeutic response. While most research on T-UCRs in CRC has focused on cell-autonomous functions, emerging evidence suggests they also act as critical modulators of the TME through several interconnected mechanisms: regulating key signaling hubs, mediating intercellular crosstalk, influencing metabolic crosstalk, and serving as potential biomarkers for TME classification (Zhang et al., 2018; Chen et al., 2024; Sakamoto et al., 2020; Kogure et al., 2013; Teresa et al., 2025; Andrea et al., 2020; Gibert et al., 2022; Wang et al., 2022; Li et al., 2018).

T-UCRs regulate several signaling pathways that have dual roles in driving cell-intrinsic oncogenesis and shaping the extrinsic TME. A prime example is the Wnt/β-catenin pathway, which is aberrantly activated in most cancers. β-catenin activation not only promotes proliferation but also induces the expression of immunosuppressive cytokines and modulates immune checkpoint molecules (Andrea et al., 2020). Research in hepatobiliary cancers has demonstrated that the expression of specific T-UCRs is directly modulated by Wnt signaling, suggesting a feedback loop where T-UCRs may amplify or stabilize Wnt pathway activity (Gibert et al., 2022). In CRC, T-UCRs like uc.285+ (which stabilizes CDC42) and uc.338 (which promotes proliferation) are implicated in pathways upstream or parallel to Wnt pathway (Zhang et al., 2018; Chen et al., 2024). Therefore, T-UCR dysregulation likely contributes to establishing a Wnt-driven, immunosuppressive TME landscape. Furthermore, T-UCRs can regulate inflammatory mediators like NF-κB, a master regulator of pro-tumorigenic inflammation that recruits immunosuppressive myeloid cells and promotes angiogenesis (Sakamoto et al., 2020). By modulating these core pathways, T-UCRs lay the foundational signaling context that dictates TME composition and function.

A potent mechanism for TME modulation is the packaging of regulatory molecules into extracellular vesicles (EVs), such as exosomes, for delivery to neighboring or distant cells (Wang et al., 2022). Compelling evidence from hepatocellular carcinoma shows that the T-UCR TUC339 is enriched in tumor-derived EVs and promotes tumor growth and adhesion upon transfer to recipient cells, establishing a direct role for a T-UCR in intercellular signaling (Kogure et al., 2013). Although specific studies on T-UCR-containing EVs in CRC are pending, this paradigm is likely conserved. For instance, EV-borne T-UCRs could be taken up by tumor-associated macrophages (TAMs), skewing them towards a pro-tumorigenic M2 phenotype, or by T cells, inhibiting their effector functions (Li et al., 2018). This represents a critical frontier for future research to understand how T-UCRs mediate cross-talk between cancer cells and the TME.

Metabolic reprogramming of the TME is a hallmark of cancer that fuels tumor growth and suppresses anti-tumor immunity (Teresa et al., 2025). Cancer cells alter nutrient availability (e.g., glucose, glutamine, choline) to create a metabolically hostile niche for immune cells. While the direct role of T-UCRs in CRC cell metabolism is not yet defined, their established connections to major oncogenic pathways position them as potential regulators of this process. For example, the Wnt/β-catenin and PI3K/AKT pathways, influenced by T-UCRs like uc.338, are key drivers of aerobic glycolysis (Zhang et al., 2018). Future investigations should explore whether T-UCRs regulate metabolic enzymes or transporters, thereby contributing to the metabolic immunosuppression characteristic of many CRC TMEs, particularly in microsatellite-stable (MSS) tumors (Teresa et al., 2025).

In conclusion, T-UCRs exhibit complex regulatory functions in the pathogenesis and progression of CRC. Comprehensive investigation into the roles and mechanisms of T-UCRs is essential for elucidating the pathophysiological processes underlying CRC and for identifying insights that may inform the development of innovative targeted therapies. Continued research in this area is anticipated to enhance our understanding of T-UCRs involvement in cancer, thereby promoting more effective clinical applications.

## 4 The mechanisms of T-UCRs in CRC

**FIGURE 4:**
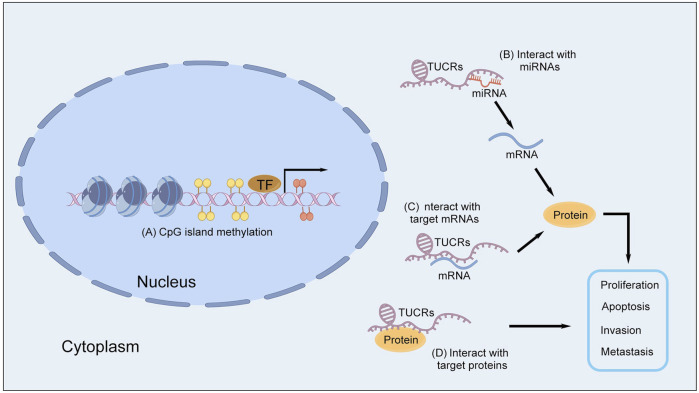
The mechanisms of T-UCRs in CRC. **(A)** CpG island methylation. **(B)** Interaction with miRNAs. **(C)** Interaction with target mRNAs. **(D)** Interaction with target proteins. The image was created using the Figdraw 2.0 (https://www.figdraw.com/).

Despite the recognized significance of T-UCRs in regulating colorectal cancer (CRC) pathogenesis, their precise mechanisms remain an area of active investigation. Recent studies suggest that T-UCRs contribute to tumor regulatory processes via several key pathways, including CpG island methylation, interactions with microRNAs (miRNAs), and direct binding to target messenger RNAs (mRNAs) or proteins (Figure 4).

### 4.1 CpG island methylation

DNA methylation refers to the addition of a methyl group to the cytosine base of DNA, leading to the formation of 5-methylcytosine. This epigenetic modification predominantly occurs at CpG dinucleotides, which are densely clustered in genomic regions known as CpG islands. These islands are typically located at the transcription start sites of genes and play crucial roles in transcriptional regulation (Stielow et al., 2021; Balasubramanian et al., 2012; Sarda et al., 2017). In normal cells, most CpG islands remain unmethylated; however, in tumor cells, a significant proportion of CpG islands exhibit aberrantly elevated methylation levels, which is intricately linked to tumorigenesis and considered a potential tumor marker (Brinkman et al., 2019; Arechederra et al., 2018; Wu et al., 2010).

Researchs indicate that while approximately half of the T-UCR-associated CpG islands remain unmethylated across various tissues, the other half demonstrates tissue-specific methylation patterns (Lujambio et al., 2010; Jones and Baylin, 2007; Lujambio and Esteller, 2007). In CRC, T-UCRs significantly modulate gene expression by influencing the methylation status of CpG islands. Aberrant methylation frequently results in silencing tumor suppressor genes or activating oncogenes (Issa, 2000). It has been demonstrated that T-UCRs can undergo methylation-related silencing within cancer cells, where methylation of promoter CpG islands plays a crucial role in the inactivation of tumor suppressor genes or genes associated with tumorigenesis (Lujambio et al., 2010).

Previous study has used both pharmacological and genomic methodologies to identified potential aberrant epigenetic silencing patterns of T-UCRs by administering DNA demethylating agents to cancer cells. Among the T-UCRs reactivated by drug treatment, uc.160+, uc.283 + A, and uc.346+ demonstrated specific silencing associated with hypermethylation of CpG islands in cancer cells compared to normal tissues. An analysis of 283 primary human tumor samples revealed that elevated methylation levels of T-UCR-associated CpG islands are widespread across various tumor types, and are detectable in early tumor lesions (Lujambio et al., 2010).

Furthermore, it was observed that the hypermethylation status of CpG islands in invasive colorectal tumors, including uc.160+, uc.283 + A, and uc.346+, is already present in precancerous lesions (Kottorou et al., 2020; Lujambio et al., 2010). In CRC specimens, such as adenomas, invasive lymph nodes, and metastatic tissues, methylation levels exhibit a progressive decline from *in situ* carcinoma to invasive and metastatic cancer stages. Notably, the methylation levels of uc.160 and uc.283 are linked to the dysplastic grading of adenoma specimens, with elevated methylation of uc.283 associated with prolonged overall survival (OS) (Kottorou et al., 2020).

A separate investigation demonstrated a marked reduction in the expression levels of uc.160, uc.283, and uc.346 in CRC tumor tissues, while methylation levels in both tumor tissues and plasma exhibited an inverse trend. The combined levels of uc.160 and the three T-UCRs in plasma are regarded as promising biomarkers (Kottorou et al., 2018). These results imply that T-UCRs may play a role in the initiation and progression of CRC through the regulation of CpG island methylation, highlighting their potential utility in epigenetic therapeutic strategies.

### 4.2 Interaction with miRNAs

The interaction between T-UCRs and microRNAs (miRNAs) constitutes a critical regulatory pathway in CRC. Empirical studies have identified direct interactions between T-UCRs and miRNAs, revealing a correlation between their expression levels. A seminal study in 2007 first demonstrates that T-UCRs are negatively regulated through direct interactions with miRNAs in chronic lymphocytic leukemia (Calin et al., 2007).

Microarray analyses have shown that the transfection of uc.283 + A in the CRC cell line HCT116 leads to differential expression of various miRNAs. Specifically, uc.283 + A influences miRNA function by binding to pri-miR-195, thereby downregulating the levels of mature miR-195 (Liz et al., 2014). Additionally, uc.77- is significantly downregulated in CRC tissues and cell lines, and its ectopic expression substantially inhibits the proliferation and growth of CRC cells *in vitro*. Mechanistic investigations reveal that uc.77- suppresses CRC cell proliferation by competing with miR-4676-5p for binding to FBXW8 mRNA through a competing endogenous RNA (ceRNA) mechanism, exerting a negative regulatory effect on CDK4. The uc.77-/miR-4676-5p/FBXW8 axis is pivotal in the pathogenesis of CRC, positioning uc.77- as a promising therapeutic target (Zheng et al., 2021).

### 4.3 Interaction with target mRNAs and proteins

Beyond their interactions with miRNAs, T-UCRs also modulate the stability, translation, or degradation of target mRNAs through direct binding. In CRC, uc.338 has been implicated in promoting cell proliferation and the G1/S phase transition by downregulating p21 and upregulating cyclin D1 (Zhang et al., 2018). In addition, uc.338 also facilitates CRC cell migration and invasion by targeting TIMP1 (Wang et al., 2016).

Recent studies have uncovered a dual-target regulatory mechanism whereby T-UCRs directly bind to both mRNAs and proteins. In CRC cells, uc.285+ facilitates cell proliferation and augments the stability of CDC42 mRNA and protein via direct binding, indicating its potential as a novel diagnostic biomarker for CRC (Chen et al., 2024).

## 5 Conclusion and future perspective

T-UCRs, as unique elements within the non-coding RNA family, play integral roles in the initiation, progression, and metastasis of CRC. While significant advancements have been made in elucidating the functions and mechanisms of T-UCRs in CRC, several limitations remain that necessitate further exploration in future investigations.

Additionally, the influence of T-UCRs expression levels on signaling pathways remains inadequately understood. Although previous research has utilized bioinformatics methods to predict potential mechanisms underlying the dysregulation of T-UCRs expression, elucidating their functions based solely on sequence or secondary structure continues to be a challenging endeavor.

Currently, the majority of research concerning T-UCRs’ functions is primarily based on *in vitro* experiments and small-scale *in vivo* studies. This underscores the necessity for further validation of their biological significance and clinical applicability. The notable conservation of T-UCRs between murine and human cells presents distinct advantages for clinical translational research. Therefore, it is essential to conduct large-scale studies involving clinical samples to comprehensively characterize the expression profiles and functional attributes of T-UCRs across various pathological contexts.

Moreover, validating the potential of T-UCRs as novel biomarkers and therapeutic targets is essential. Such studies are anticipated to provide new insights into the diagnosis and prognosis of CRC, establishing a foundation for the development of personalized therapeutic strategies.

In summary, this review mphasizes that T-UCRs are not merely bystanders but pivotal regulators in CRC, operating through interconnected networks that control cell fate, modulate the tumor microenvironment, and are frequently silenced by epigenetic mechanisms. Enhancing our understanding of T-UCRs in CRC is crucial for developing innovative diagnostic and therapeutic strategies. Continued research should connect basic science with clinical practice to improve CRC patient outcomes.
